# Supplementary material for: Downregulation of IL-8 and IL-10 by the Activation of Ca2+-Activated K+ Channel KCa3.1 in THP-1-Derived M2 Macrophages
Source: Int J Mol Sci. 2022 Aug 3;23(15):8603. doi: 10.3390/ijms23158603 (PMC9368915; doi:10.3390/ijms23158603)
Supplement: Supplementary file 1 [file ijms-23-08603-s001.zip › ijms-1851898-supplementary.pdf]

Supplementary Materials: The following are available online at www.mdpi.com/xxx/s1.

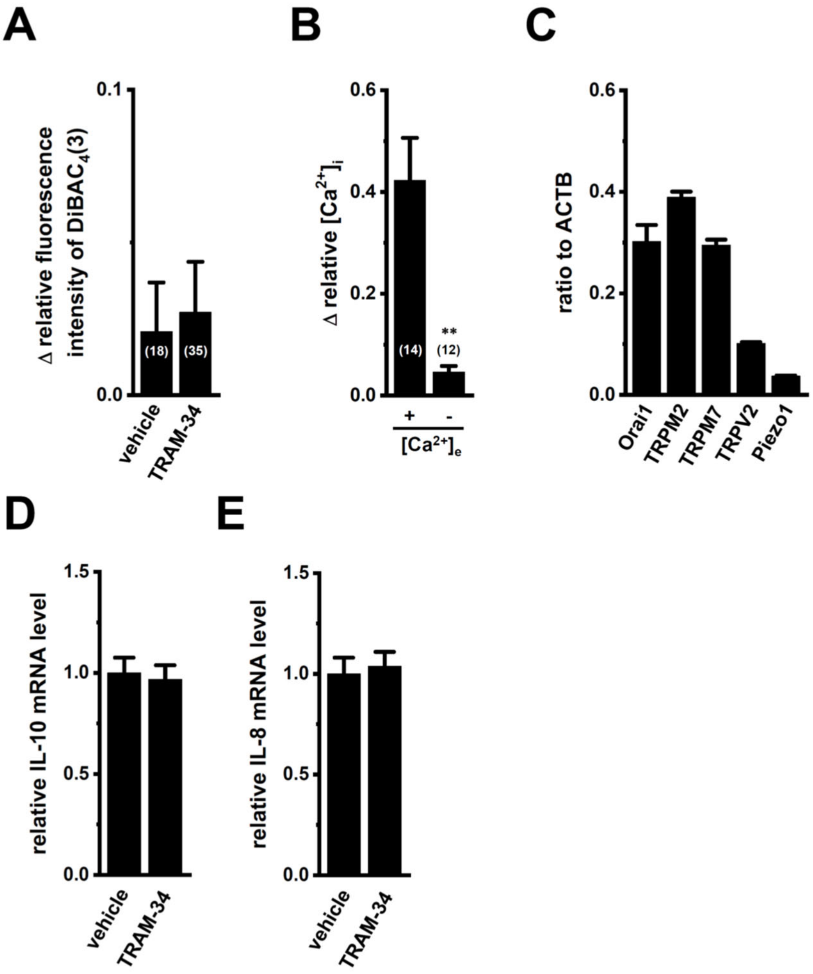

Figure S1. Effect of TRAM-34 on the membrane potential, SKA-121-induced $[Ca^{2+}]_i$ change under Ca^{2+}-free condition, expression levels of Ca^{2+}-permeable channels, and effect of the treatment with TRAM-34 alon for 24-hr on the expression levels of IL-10 and IL-8 transcripts in THP-1-derived M_2 macrophages. A: TRAM-34 (1 μM)-induced changes in the relative fluorescence intensity of $DiBAC_4(3)$ in THP-1-derived M_2 macrophages. Numbers used for experiments are shown in parentheses ($p > 0.05$ vs. the vehicle control). B: SKA-121 (1 μM)-induced changes in $[Ca^{2+}]_i$ in the absence (-) or presence (+) of Ca^{2+} in the extracellular solution ($[Ca^{2+}]_e$) in THP-1-derived M_2 macrophages. Numbers used for experiments are shown in parentheses (**: $p < 0.01$ vs. + $[Ca^{2+}]_e$). C: Real-time PCR examination of Orai1, TRPM2, TRPM7, TRPV2, and Piezo1 expression in THP-1-derived M_2 macrophages. Expression levels are shown as a ratio to ACTB (n = 4 for each). D, E: Effects of the treatment with TRAM-34 (10 μM) for 24-hr on the expression levels of IL-10 (D) and IL-8 (E) transcripts in THP-1-derived M_2 macrophages. The expression level in the vehicle control was expressed as 1.0 ($p > 0.05$, n = 4 for each).

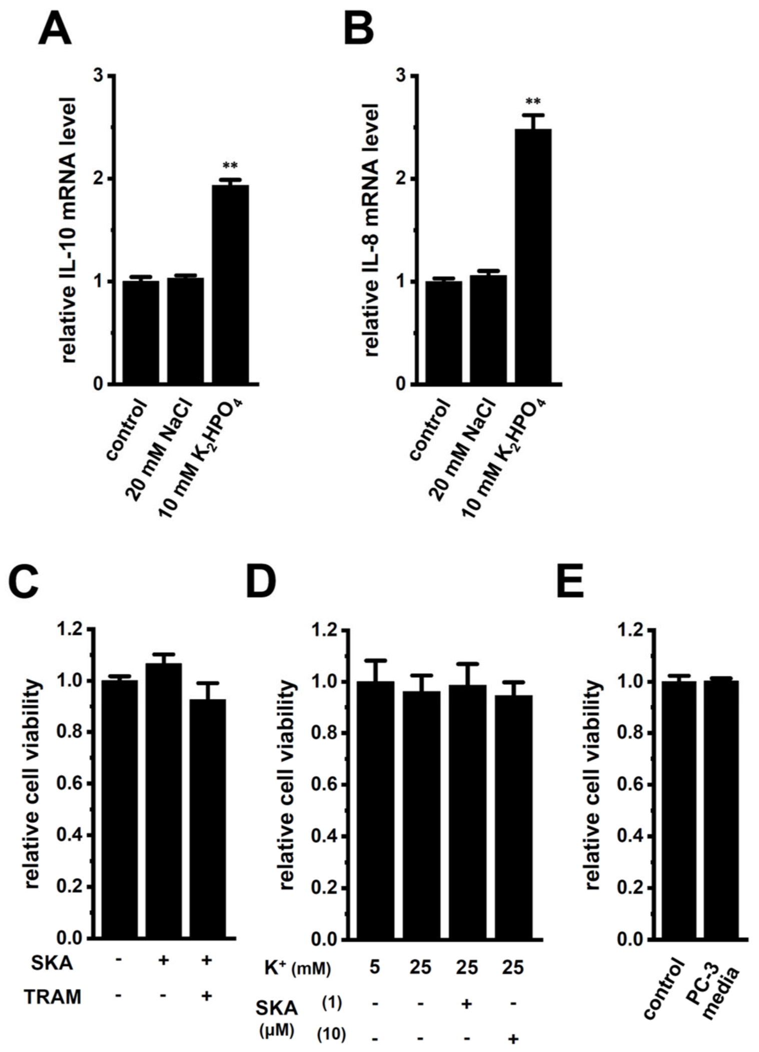

Figure S2. Effects of treatments with 20 mM NaCl and 10 mM K_2HPO_4 on expression levels of IL-10 and IL-8 transcripts and effects of treatments with 1 μM SKA-121, high $[K^+]_e$ exposure, and PC-3 media on the viability of THP-1-derived M_2 macrophages. A, B: Real-time PCR examination of IL-10 (A) and IL-8 (B) expression in 20 mM NaCl and 10 mM K_2HPO_4-treated THP-1-derived M_2 macrophages for 24 hr. Relative mRNA expression in normal $[K^+]_e$ (control) is expressed as 1.0 (n = 4 for each). C-E: Effects of the treatment with 1 μM SKA-121 and/or 10 μM TRAM-34 (C), the exposure to high $[K^+]_e$ (25 mM) in the absence and presence of 1 or 10 μM SKA-121 (D), and PC-3 media (E) for 24 hr on the viability of THP-1-derived M_2 macrophages. Cell viability in the vehicle control (-/- in 'C'), normal $[K^+]_e$ (5 mM K^+ in 'D'), or normal culture media (control in 'E') is expressed as 1.0 (n = 5 for each). **: *p* < 0.01 vs. the control.

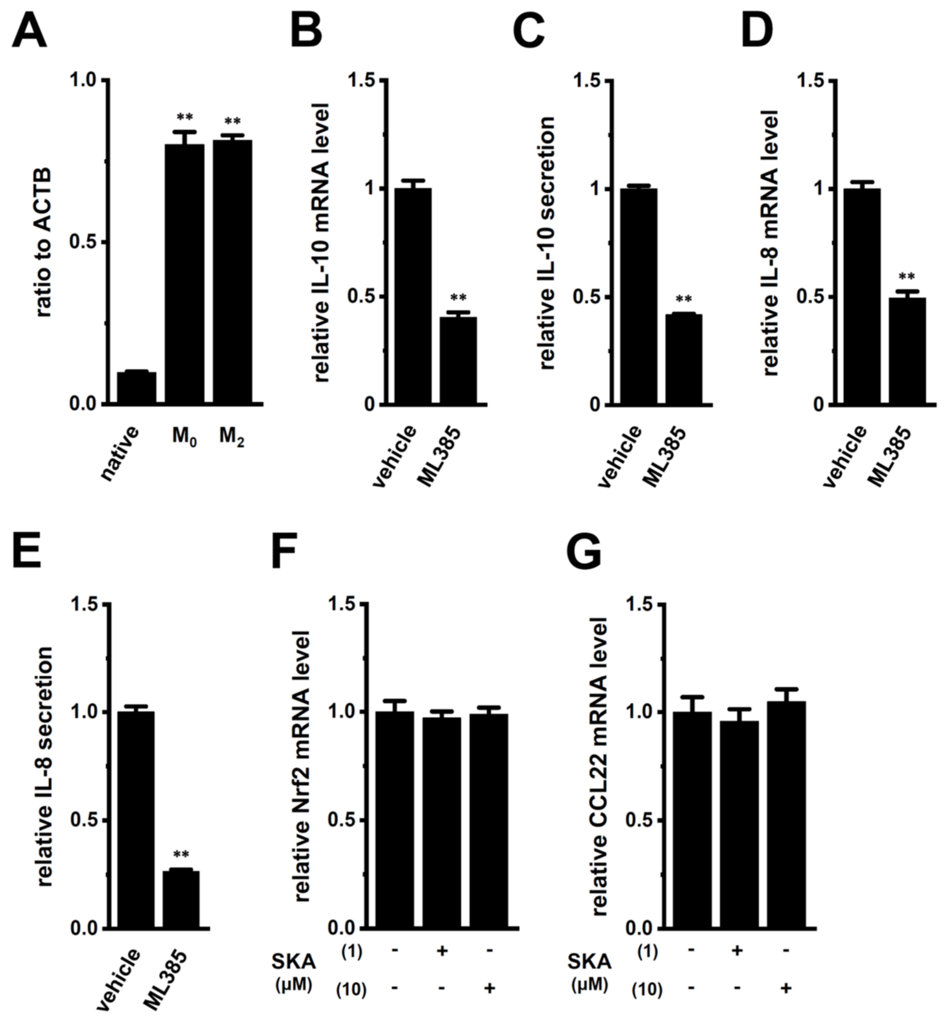

Figure S3. Effects of a treatment with a Nrf2 inhibitor on IL-10 and IL-8 expression and secretion and effects of a treatment with SKA-121 on Nrf2 and CCL22 expression in THP-1-derived M_2 macrophages. A: Real-time PCR examination of Nrf2 expression in native THP-1 ('native'), THP-1-derived M_0 macrophages ('M_0'), and THP-1-derived M_2 macrophages ('M_2'). Expression levels are shown as a ratio to ACTB (n = 4 for each). B, D: Real-time PCR examination of IL-10 (B) and IL-8 (D) expression in vehicle- and ML385 (10 μM)-treated THP-1-derived M_2 macrophages for 24 hr. C, E: Quantitative detection of IL-10 (C) and IL-8 (E) secretion by an ELISA assay in vehicle- and ML385 (10 μM)-treated THP-1-derived M_2 macrophages for 24 hr (n = 4 for each). F, G: Real-time PCR examination of Nrf2 (F) and CCL22 (G) expression in THP-1-derived M_2 macrophages treated (+) or untreated (-) with 1 and 10 μM SKA-121 for 24 hr. Relative mRNA expression in the vehicle control (-/-) is expressed as 1.0 (B, D, F, G) (n = 4 for each). **: $p < 0.01$ vs. 'native' and the vehicle control.
